# The retrovirus RNA trafficking granule: from birth to maturity

**DOI:** 10.1186/1742-4690-3-18

**Published:** 2006-03-17

**Authors:** Alan W Cochrane, Mark T McNally, Andrew J Mouland

**Affiliations:** 1 Department of Medical Genetics and Microbiology, University of Toronto, 1 King's College Circle, Toronto, Ontario, M5S 1A8, Canada; 2 Department of Microbiology and Molecular Genetics, Medical College of Wisconsin, Milwaukee, WI, 53226, USA; 3 HIV-1 RNA Trafficking Laboratory, Lady Davis Institute for Medical Research-Sir Mortimer B. Davis Jewish General Hospital and McGill University, 3755 Côte-Ste-Catherine Road, H3T 1E2, Canada

## Abstract

Post-transcriptional events in the life of an RNA including RNA processing, transport, translation and metabolism are characterized by the regulated assembly of multiple ribonucleoprotein (RNP) complexes. At each of these steps, there is the engagement and disengagement of RNA-binding proteins until the RNA reaches its final destination. For retroviral genomic RNA, the final destination is the capsid. Numerous studies have provided crucial information about these processes and serve as the basis for studies on the intracellular fate of retroviral RNA. Retroviral RNAs are like cellular mRNAs but their processing is more tightly regulated by multiple *cis*-acting sequences and the activities of many *trans*-acting proteins. This review describes the viral and cellular partners that retroviral RNA encounters during its maturation that begins in the nucleus, focusing on important events including splicing, 3' end-processing, RNA trafficking from the nucleus to the cytoplasm and finally, mechanisms that lead to its compartmentalization into progeny virions.

## Background

The life of an mRNA is directed by the protein components of ribonucleoprotein particles (RNP) whose roles include nuclear processing reactions, transport, translation and degradation. Retroviral replication depends on many of the same processes to form viral mRNA and genomic RNA providing an experimentally tractable system to study the *cis *and *trans *determinants of mRNA fate. In this review, we summarize the current understanding of the processes affecting retroviral RNA metabolism as the RNA moves from its site of synthesis within the nucleus to its encapsidation into viral particles that emerge from the plasma membrane. The field has not only illuminated the cellular processes regulating RNA fate in general but also provided insights into potential strategies to impair replication of these viral pathogens.

## Preserving genome-length RNA

### Splicing control – the role of the NRS of ASV

**Figure 1:**
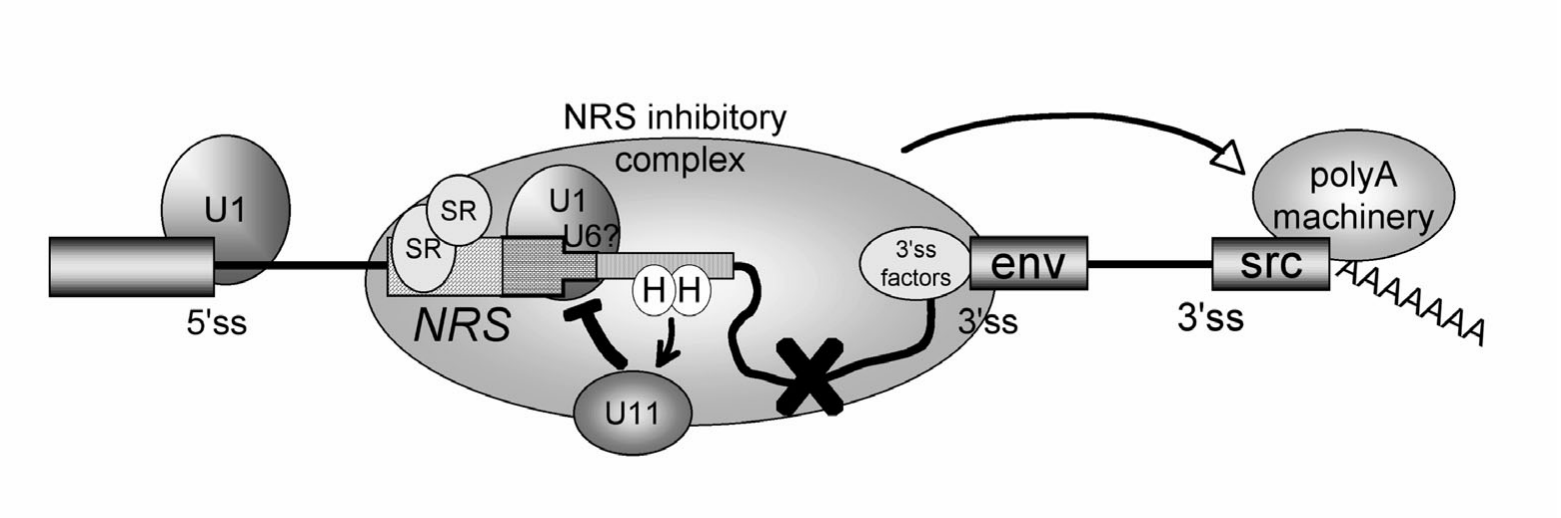
**Model for NRS effects on splicing and polyadenylation**. Schematic of RSV RNA with exons depicted as boxes and introns shown as thin lines. The light shading represents the upstream SR protein binding region of the bipartite NRS, and the darker shading depicts the region that binds U1 snRNP. SR proteins promote U1 binding, which initiates early interactions with factors associated with the viral 3' splice site (env in this example), and this is thought to mature into a spliceosome-like NRS inhibitory complex (indicated by the large oval) that forms between the NRS and the viral 3' splice site but which is catalytically inactive (an X over the intron); a possible role for U6 snRNP is indicated by the question mark. The NRS complex sequesters the 3'ss from interacting with the authentic viral 5'ss to block splicing. The NRS complex may influence polyadenylation by serving to stabilize the binding of splicing factors to the weak viral 3' splice site, which can then either recruit or stabilize the polyadenylation complex (arrow) and thereby enhance polyadenylation of viral unspliced RNA. U11 snRNP modulates NRS function by antagonizing U1 binding and assembly of the NRS inhibitory complex. A downstream region (intermediate shading) binds hnRNP H, which recruits U11 to a site that overlaps the U1 binding site.

The expression of viral proteins from unspliced, incompletely spliced and fully spliced transcripts has necessitated that retroviruses evolve strategies to control the extent of RNA splicing. Extensive studies of avian sarcoma virus (ASV) splicing revealed three mechanisms of splicing control. The first involves the maintenance of suboptimal 3' splice site (ss) signals. Use of the *env *3'ss is controlled by a suboptimal branchpoint (bpt) sequence and a nearby exonic splicing enhancer (ESE) [1,2] whereas the *src *3' ss has a suboptimal pyrimidine tract (ppt) [3]. Mutations that improved the quality of the signals increased splicing and had detrimental effects on replication. Consistent with a requirement of inefficient splicing for optimal replication, revertants contained mutations that restored inefficient splicing. In addition to suboptimal splicing signals, a second, poorly characterized negative element is also present upstream of the *src *3' ss [4,5]. Whether this element represents an intronic splicing silencer (ISS) and what factors bind to it remains to be determined. These two splicing control mechanisms are shared with HIV (discussed below). A third, novel control element in Rous sarcoma virus (RSV), that is apparently unique to avian retroviruses, is the negative regulator of splicing, or NRS [6,7]. The NRS is thought to represent an elaborate pseudo-5'ss that non-productively interacts with and sequesters the viral 3' splice sites such that productive splicing with the authentic 5'ss cannot occur (Figure 1). In addition to its established role in splicing control, the NRS serves a second function in promoting efficient polyadenylation of viral transcripts, as discussed below.

The NRS was originally identified from *gag *intron deletion mutations that increased splicing in RSV [6-8]. The ability of the NRS to block splicing of heterologous introns facilitated elucidation of factors that bind to it and its mechanism of action [6]. Unlike other negative elements in HIV and RSV that are close to 3' splice sites, the ~230 nt NRS is located in the *gag *intron approximately 300 nt from the 5'ss and more than 4000 nt from the first of two alternative 3' splice sites (*env *and *src*) [9]. Mutagenesis studies determined the NRS to be bipartite; splicing inhibition requires a diffuse upstream purine-rich element separated by ~115 nt from a discrete downstream sequence that resembles a conventional but degenerate U1-type 5' ss [9-12]. Overlapping the degenerate U1-type 5'ss is a consensus binding site for U11 snRNP, a factor that serves an analogous role to U1 in binding the 5'ss of a rare class of introns that are spliced by a second, low abundance spliceosome [13]. Binding of both U1 and U11 to the NRS has been demonstrated however it is the interaction with U1 that leads to splicing inhibition [10,11,14]. The mechanism by which U1 binding to the NRS leads to inhibition rather than NRS splicing is not clear, but may involve an aberrant U6 interaction at a later step (M.T.M., unpublished). The U1/U11 sites overlap and thus binding is mutually exclusive. U11 binding may regulate splicing inhibition by modulating U1 binding, and contribute to the balance of unspliced to spliced RNA and replication. Thus, determining the *cis *and *trans *factors that govern U1 and U11 binding was important.

The upstream, purine-rich region of the NRS was shown to have potent splicing enhancer activity and to bind members of the SR protein family of splicing factors and hnRNP H [11,15,16]. One function of splicing enhancers and SR proteins is recruitment of components of the splicing apparatus. In the case of the NRS, it was shown that the role of the enhancer region and SR proteins was to recruit U1 to the downstream degenerate 5'ss. In contrast, the SR protein-binding region was not necessary for efficient U11 binding [11]. The NRS itself forms an early spliceosome-like complex that is dependent on U1 and SR proteins, and this complex can interact with a 3'ss in a U1-dependent manner [17-19]. This interaction persists into an ATP-dependent, more mature splicing-like complex, however this complex is distinguished from authentic splicing complexes in that the U4:U6/U5 tri-snRNP is not stably bound and the U5-associated protein Prp8 cannot be cross-linked to the 5' ss [198]. It is this aberrant snRNP association that presumably accounts for assembly of a non-catalytic complex that leads to sequestration of the viral 3' ss and culminates in splicing inhibition.

The determinants for U11 binding are largely distinct from U1. U11 is at a competitive disadvantage for NRS binding, being 100-fold less abundant than U1 [20]. It was recently shown that optimal U11 binding requires an upstream 3'ss-like sequence and a downstream G-rich region [21]. The downstream G-rich region binds hnRNP H, and mutations in the G-tracks or depletion of hnRNP H reduces U11 binding in vitro and in vivo [22]. These lessons from RSV suggested a more general role for hnRNP H in U11 binding and splicing of authentic minor-class introns. Indeed, the SCN4A and P120 minor-class introns have G tracts, bind hnRNP H, and require hnRNP H for optimal splicing [22]. HnRNP H also plays a role in U1 binding to an HIV-1 enhancer [23], which is consistent with recent demonstrations that splicing of some U2-dependent introns requires hnRNP H [24,25].

### HIV-1 splicing

**Figure 2:**
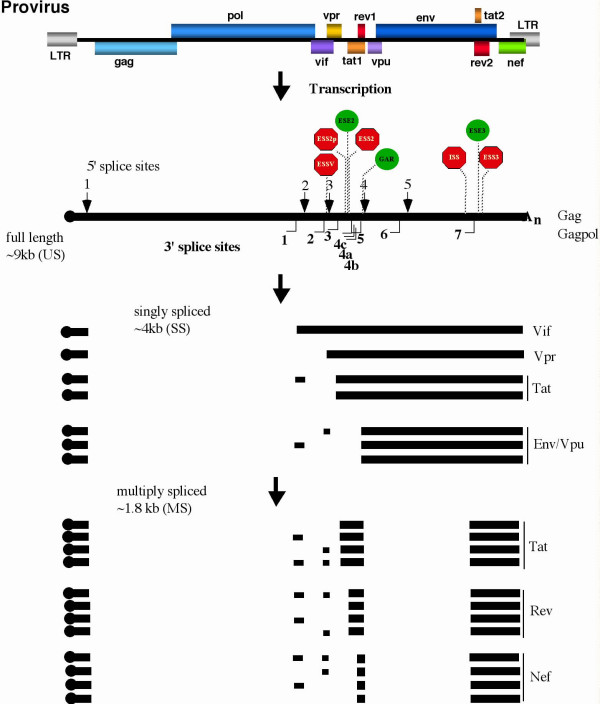
**Processing of HIV-1 RNA**. Outlined in the figure are the cis-acting components of the HIV-1 RNA which control its processing. Indicated are the positions of the 5' splice sites (arrows above the unspliced RNA), 3' splice sites (brackets below the unspliced RNA), and the various ESS and ESE elements that modulate splice site use. At top is an outline of viral genome and on the bottom, the exons which comprise the major spliced forms (4.0 kb singly spliced and 1.8 kb multiply spliced) of the genomic RNA are indicated by black boxes. Multiple spliced RNAs combining or excluding various exons encode each of the viral accessory proteins.

In contrast to murine leukemia and avian sarcoma viruses, the increased coding capacity of HIV-1 has necessitated the evolution of a more complex splicing regimen. In addition to structural proteins, HIV-1 expresses six additional proteins that regulate various facets of the virus lifecycle [26]. To account for this increased coding potential, the 9 kb HIV-1 transcript is processed into over 30 mRNAs through alternative splicing [27,28]. The products are grouped into three size classes: the unspliced, 9 kb RNA encoding Gag and Gag/Pol, the 4 kb, singly spliced RNAs that encode Vif, Vpr, Vpu and Env, and the 2 kb, multiply spliced RNAs that express Tat, Rev and Nef. Generation of the required viral RNAs is achieved through the combinatorial use of five 5' splice sites (SD1-5) and nine 3' splice sites (ss) (SA1-3, SA4a,b,c, SA5-SA7). The production of a spectrum of RNAs from unspliced to multiply spliced necessitated the development of multiple mechanisms to control the extent of viral RNA splicing since a substantial amount of unspliced RNA is needed for replication. Initial analysis of HIV-1 RNA processing focused on the splice sites themselves and demonstrated that while the 5'ss were highly active, the 3'ss were suboptimal due to alterations in either the ppt or bpt sequences [29-31] (Figure 2).

Subsequent research determined that the suboptimal nature of the 3'ss of HIV-1 was not the only point of regulation. It was established that exon sequences also influence the use of individual 3'ss. These exon regulatory sequences fall into two groups; exon splicing enhancers (ESEs) that act to enhance recognition and use of the adjacent splice site, and exon splicing silencers (ESSs) that suppress the use of adjacent 3'ss. To date, four ESSs have been mapped and control the use of the 3'ss for Vpr (ESS-V), Tat (ESS2, ESS2p), and the terminal 3'ss (ESS3) [30,32-37]. For ESS-V, ESS2, and ESS3, function is dependent upon an interaction with members of the hnRNP A/B protein family [34,38-40] that results in an early block to spliceosome formation. In the case of ESS3, initial work suggested that binding of hnRNP A1 to ESS3 initiates oligomerization of hnRNP A1 along the RNA, sterically hindering recognition of the ppt and bpt sites by the corresponding splicing factors [41]. Subsequent analyses suggested an alternative mechanism and the involvement of an intronic splicing silencer (ISS) to which hnRNP A1 also binds [42,43]. Multiple hnRNP A1 binding sites have also been mapped within ESS3 [42]. Mutations that disrupt hnRNP A1 binding to either the ISS or ESS3 result in partial alleviation of inhibition and mutation of both is more severe [40], suggesting hnRNP A1 proteins, bound at ESS3 and ISS, might interact to loop out the intervening sequence and impair splicing factor binding to the bpt and ppt. Such a looping mechanism involving hnRNP A1 binding to separate sites has been proposed for regulation of exon 7B in the hnRNP A1 pre-mRNA [44,45]. Function of ESS2p is less well studied but correlates with hnRNP H binding [35].

Countering the inhibitory signals of the ESSs are the three ESEs present within the first (ESE2, GAR) and second (ESE3) coding exon of Tat [32,37,46-50]. Through interaction with one of several members of the SR protein family, the ESEs act by facilitating the recruitment to and/or stabilization of factors that bind the adjacent 3'ss [51-54]. Overexpression of SF2/ASF leads to enhanced use of SA2 and to a lesser extent SA1 [55,56], and increased expression of SC35 and SRp40 augment use of SA3, presumably by blocking hnRNP A1 binding to the adjacent ESS2 [55,56]. A similar competition model was suggested to explain the countering activities of ESE3 and ESS3 that affect SA7 use [42,48,49]. In contrast to ESE2 and ESE3, the enhancer downstream of SA5 (GAR) appears to be more complicated. The 5' portion of this bipartite ESE binds SF2/ASF and the 3' half interacts with SRp40 [46]. Point mutations within GAR that abrogate factor binding to either domain reduce the efficiency of this element. In addition to promoting SA5 use, this ESE also functions in the recognition of the downstream 5'ss (SD4) by U1 snRNP. Inactivation of the GAR enhancer results in a dramatic increase in the ligation of SD1 to SA7, bypassing all of the splice acceptors used to produce the viral regulatory protein mRNAs (SA3, SA4a-c, SA5) [46]. Therefore, this GAR enhancer appears to play a critical role in ensuring the correct processing of HIV-1 RNA. As one indication of the delicate balance required to achieve the needed levels of the various viral RNAs, a point mutation within *env *results in generation of aberrant spliced products due to the creation of a splicing enhancer that activates a cryptic 3'ss (SA6) [23,57].

### Control of splicing in other retroviruses

While RNA processing has been most extensively studied in ASV and HIV-1, work in other systems has also illuminated patterns of splicing regulation. Studies of equine infectious anemia virus (EIAV) have identified both *cis *and *trans *modulators of RNA processing. Examination of EIAV splice sites revealed that both the 5'ss and bpt sequences do not deviate significantly from consensus. In contrast, the polypyrimidine tracts are interrupted by purines, which may reduce splice site usage by decreasing binding of the splicing factor U2AF [58]. The purine-rich element (PRE) that comprises the EIAV Rev (eRev) binding site is also involved in splicing regulation [59,60]. Deletion of the PRE results in a marked increase in unspliced and Tat-encoding RNAs and a reduction in eRev RNA [58]. This observation suggests that the PRE acts like an ESE to promote adjacent splice site use. In vitro experiments demonstrated that SF2/ASF can bind the PRE [60,61]. However, overexpression of SF2/ASF failed to promote use of the splice site adjacent to the PRE but rather increased the level of unspliced viral RNA and reduced the quantity of eRev RNA. In parallel experiments, hnRNP A1 overexpression failed to alter viral RNA splicing patterns [58].

In contrast to HIV-1, there is only a limited understanding of splicing control in human T cell leukemia virus type 1 (HTLV-1). Like HIV-1, HTLV-1 produces several factors, in addition to the structural proteins, by alternative splicing. Little is known of the cis-acting elements controlling splice site use but evidence of regulation is provided by the marked differences in abundance of the various spliced RNA isoforms in different infected cell lines [62,63]. Overexpression of SF2/ASF or hnRNP A1 alters HTLV-1 RNA splicing patterns [63] and loss of hnRNP A1 expression leads to an accumulation of unspliced viral RNA and increased virus production [64]. Although the effect of hnRNP A1 depletion could be attributed to affects on splicing, the data could also be explained if hnRNP A1 inhibits viral RNA transport to the cytoplasm (putatively by inhibiting binding of the HTLV-1 Rev-like factor, Rex, to the viral RNA) [65].

Several cis-acting elements that affect RNA stability and processing in Moloney murine leukemia virus (MoMLV) have also been identified. Analysis of sequences adjacent to the 3'ss revealed several elements that control splicing [66]. Deletion of exon sequences downstream of the 3'ss resulted in a total loss of spliced viral RNA, suggesting that the region may contain an ESE as seen for EIAV, HIV-1 and ASV. In contrast, deletion of 140 nt immediately upstream of the bpt sequence resulted in a marked elevation in the spliced/unspliced viral RNA ratio, consistent with the presence of a splicing silencer. However, the region surrounding the 3'ss is not the only one that influences splicing. Another element located within the CA region is required for accumulation of spliced viral RNA [67,68]. This element contrasts with the NRS of ASV as the MoMLV element would appear to be a stimulator of viral RNA splicing. Studies on the Akt strain of MLV identified a region downstream of the 5'ss that modulates splicing efficiency. In the course of examining the contribution of various sequence elements to viral RNA dimer initiation, Aagaard et al. [69] noted that deletion of a stem loop structure (DIS-1) immediately 3' of the 5'ss resulted in a 5–10 fold increase in the level of spliced RNA. Given its close proximity to the 5'ss, the secondary structure of DIS-1 may block base pairing of U1 snRNA to the 5' ss.

In summary, it would appear that retroviruses have used a common set of tools (suboptimal 3'ss, splicing enhancers, splicing silencers) to regulate the extent of viral RNA processing and achieve a balanced level of unspliced and spliced RNAs compatible with virus replication. The use of cellular factors (SR proteins, hnRNP proteins) to regulate splicing suggests that the extent of RNA processing and hence, the capacity of the virus to replicate, is also dictated by the required mix of host cell splicing regulatory factors and determine the host range of the virus. Supporting this conclusion are observations from studies using MLV and HIV-1 based vectors. Lee et al. [70] noted that virus titers from a MLV-based vector varied significantly between various cell lines and showed that at least part of the problem resided in marked differences in viral RNA processing. Since the vector used was constant, the variation seen is likely due to different levels of host splicing factors. A similar phenomenon may also partially explain the inability of murine cells to support HIV-1 replication. Zheng et al. [71] observed excessive splicing of HIV-1 RNA upon introduction of provirus into murine cells. This problem could be alleviated by expression of the human p32 protein, which binds to and likely sequesters SF2/ASF. By reducing the availability of SF2/ASF in this manner, the extent of HIV-1 RNA splicing is reduced, permitting accumulation of genomic RNA. These findings highlight the vulnerability of retroviruses to modulation of host factor regulating RNA processing and raise the possibility of therapeutic intervention at this level.

### Polyadenylation

Polyadenylation plays a key role in the life of an mRNA, regulating its transport, translation and turnover. Controlling where in the retroviral genome polyadenylation occurs is critical for replication. For several retroviruses (i.e. HTLV-1, HTLV-2, bovine leukemia virus, RSV, murine leukemia virus), choice of polyadenylation site use is straightforward since the major signal for the reaction (AAUAAA) occurs only once in the transcript. For other retroviruses (i.e. HIV-1, equine infectious anemia, Moloney murine leukemia virus), the situation is rendered more complex by the duplication of the polyadenylation signals (AAUAAA and the 3' G/U-rich sequence) at the 5' and 3' ends of the transcript. Successful replication has necessitated that these viruses evolve mechanisms to suppress recognition of the first polyadenylation signals. Although research has shown that sequences within U3 (present only at the 3' end of the retroviral RNA) can enhance use of the downstream polyadenylation signal, this finding does not readily explain its almost exclusive use.

### Regulating HIV-1 RNA polyadenylation

In the case of HIV-1, it was initially believed that the proximity of the first polyadenylation site to the start site of transcription reduced its recognition by the host polyadenylation machinery, possibly as a result of a secondary structure that masks the AAUAAA polyadenylation signal [72,73]. However, subsequent work has provided an alternative explanation. Inactivation of the first 5'ss (SD1) dramatically increased use of the promoter proximal polyadenylation signal [74-76]. Subsequent experiments determined that recruitment of U1 snRNP to SD1 acts to suppress use of this polyadenylation site, possibly through an interaction between the U1 70 K protein of U1 snRNP and components of the polyadenylation machinery, in particular polyA polymerase [75,77].

In addition to the cis-acting signals that control use of the first polyadenylation site, ESE3 and ESS3 play a role in modulating use of the second polyadenylation signal. Deletion of ESE3 not only results in decreased use of SA7 but also an inhibition of Rev-dependent viral gene expression [40,78] that correlated with loss of polyadenylation of the incompletely spliced viral RNA [79]. Polyadenylation of viral RNA could be restored by the deletion of ESS3 and ESE3, indicating that these two elements not only play antagonistic roles in the recognition of SA7 but also in the 3' end processing of viral RNA [79].

Cellular and viral proteins have been implicated in regulating HIV-1 RNA polyadenylation. Experiments with Sam68, a member of the STAR family of RNA binding proteins, revealed that its overexpression dramatically enhanced Rev function [80-83], which correlated with the ability to stimulate 3' end processing of incompletely spliced viral RNA [79]. With the demonstration that Sam68 is essential for both Rev-induced viral gene expression and HIV-1 replication, it would appear that it might play a pivotal role in the processing of the incompletely spliced viral RNAs that renders them competent for transport to the cytoplasm and subsequent translation [84]. Interestingly, the virus itself also modulates the cell's polyadenylation machinery. Vpr-expressing viruses induce a dephosphorylation of poly A polymerase, the enzyme responsible for addition of the poly A tail following cleavage, an alteration that leads to increased activity [85]. While Vpr expression does lead to a modest increase in viral RNA levels, this is not achieved through changes in either viral RNA stability or poly A tail length. However, it remains to be determined whether Vpr might affect the initial processing of the viral transcripts in the nucleus. HIV-1 Tat may also impact on viral RNA polyadenylation through its ability to increase expression of the 73 kDa component of the cleavage and polyadenylation specificity factor (CPSF), a key factor in 3'end processing [86].

### MLV: different virus, different solution

Although both HIV-1 and Moloney murine leukemia (MoMLV) virus share the same problem of suppressing use of the 5' proximal polyadenylation signal, they have evolved different mechanisms to solve the problem. In contrast to HIV-1, mutation of the 5'ss in MoMLV has little effect on the use of the first polyadenylation signal. Rather than regulating use of the signal, MoMLV appears to have adopted the use of inefficient signals, resulting in a significant proportion of viral transcripts failing to use either of the two viral encoded polyadenylation signals and the RNA terminates in the adjacent cellular sequences [87].

### RSV, the NRS, and 3'-end formation

In the avian RSV, the NRS appears to play an important role in modulating polyadenylation efficiency. Avian retroviral 3'-end formation is inherently inefficient with ~15% of RNA representing read-through transcripts where poly(A) addition occurs at downstream cellular sites [88]. Miller and Stoltzfus [89] showed that deletions encompassing the NRS increased the level of read-through transcripts and proposed that the deleted sequence(s) bind factors that stabilize the poly(A) machinery to allow more efficient polyadenylation. The NRS appears to be the relevant element since specific mutations or deletions within this region also result in 3'-end formation deficiencies [8,90]. It was proposed that the stalled splicing complex between the NRS and viral 3'ss serves the same function as the splicing process [90]. This model is consistent with observations that NRS mutations induce transcriptional read-through and splicing into the cellular myb gene in chickens, which results in short-latency lymphomas [91]. It will be important in the future to determine the mechanism by which the NRS boosts polyadenylation of genomic RNA.

### Nuclear export of incompletely spliced RNA

Once the challenges of manipulating the splicing apparatus to preserve pools of unspliced RNA have been met, retroviruses face the task of exporting these molecules in a cell that normally restricts unspliced RNA to the nucleus. This could involve overcoming nuclear retention signals and/or recruiting export factors that otherwise would have little attraction for genome-length viral RNA. Export of bulk mRNA is thought to be facilitated by the recruitment of the general export factor TAP/NXF1:p15 to the RNA through different adaptor proteins, including REF/Aly and SR proteins [92]. Adaptor loading onto RNA occurs either through an interaction with a mark deposited upon intron removal, such the exon junction complex (EJC), or by direct binding to elements within the RNA. How then do unspliced retroviral RNAs, which don't benefit from the deposition of an EJC, get efficiently exported? As with splicing and polyadenylation, different viruses have evolved distinct mechanisms to export unspliced RNA. In the case of HTLV-1/2 and lentiviruses such as HIV-1, the virus encodes an accessory protein that targets unspliced RNA to an export pathway distinct from that used by most cellular mRNA. In contrast, simple retroviruses like Mason Pfizer monkey virus (MPMV) harbor cis-elements that bind host cell export factors directly, independent of splicing. Moreover, some of these proteins are multifunctional, acting early in splicing regulation and later in RNA trafficking and perhaps viral RNA encapsidation.

### Control of HIV-1 RNA export out of the nucleus

Early mutagenesis studies of HIV-1 revealed that loss of Rev expression resulted in a complete loss of viral structural protein expression without significantly affecting levels of the various viral RNAs. Subsequent fractionation studies determined that the absence of HIV-1 structural protein production upon inactivation of the Rev reading frame was due to sequestration of the unspliced 9 kb and singly spliced 4 kb viral RNAs in the nucleus [93-96]. Only the fully processed 2 kb viral mRNAs accumulate in the cytoplasm in the absence of Rev. The basis for the nuclear retention of the 9 kb and 4 kb HIV-1 RNAs remains poorly understood, with some groups attributing it to partial assembly of spliceosomes on the RNA [97], while others have identified cis-acting repressive (CRS) or instability (INS) sequences within the Gag, Pol and Env reading frames that are able to confer Rev-dependency in heterologous contexts [98-107]. As these inhibitory sequences are removed by splicing in the generation of the multiple spliced, 2 kb RNAs, no impediment exists for the transport of these viral RNAs via the general mRNA export pathway of the cell. Additional mutations also demonstrated the requirement of a 240 nt sequence (designated the Rev-responsive element, RRE) within the *env *reading frame for Rev function [95,96]. The RRE serves as a point of interaction of viral RNA with the Rev protein.

Intense investigation of HIV-1 Rev function has resulted in it being one of the most thoroughly characterized export systems and readers are referred to more extensive reviews on its function that are briefly summarized here [95,96]. Multiple domains are required for Rev to function. Within the amino terminal portion of Rev is an arginine-rich stretch between a.a. 35–50 that comprises a nuclear/nucleolar localization signal (NLS/NoLS) and forms an alpha helix able to bind in the major groove of the primary Rev-binding site of RRE RNA. Within the carboxyl terminal portion is a leucine-rich sequence between a.a. 73 and 84 that forms the nuclear export signal (NES). Despite steady-state accumulation in the nucleolus, the presence of both an NLS and NES within Rev results in the protein constantly moving between the nucleus and cytoplasm. Nuclear import is mediated by binding of the arginine-rich region to the transport mediator importin β and nuclear export is achieved through binding of Crm1/Exportin-1 to the leucine-rich NES of Rev in a Ran/GTP dependent manner. This ternary complex (Rev/Crm1/RanGTP) then interacts with the FG-repeats of nucleoporins and the complex moves through the nuclear pore. Once within the cytoplasm, the RanGTP within the complex is hydrolyzed to RanGDP by RanGAP and the ternary complex disassembles.

Although it is possible that Rev interacts with all RRE-containing HIV-1 RNAs in the nucleus (the 9 and 4 kb class of RNAs), studies have indicated that several parameters dictate which RNA will be exported to the cytoplasm. First was the demonstration that Rev function was dependent upon the continued transcription of the target RNA despite the presence of significant levels of RRE-containing RNA in the nucleus [108]. This finding suggests that Rev must act before the viral RNA either becomes fully spliced or is committed to retention in the nucleus. Second was the observation that Rev-induced export required 3' end processing of the RNA as only polyadenylated viral RNAs are transported to the cytoplasm [79,109]. Therefore, while Rev is able to bypass the cellular mechanisms that prevent export of incompletely spliced RNAs from nucleus, the affected RNAs must meet a limited set of criteria (5' cap, 3' poly A tail) to be exported. The requirement for 3' end processing for Rev-mediated export may provide a partial explanation for the need for continued synthesis of the target RNA. Recent studies have established that a tight coupling exists between the various processing steps leading to mature mRNAs, suggesting that once 3' end formation occurs it would stimulate the removal of the upstream intron [110-112]. Therefore Rev may need to act within the brief time frame between 3' polyadenylation and subsequent splicing of the RNA to induce export of the unspliced and partially spliced viral RNAs. The population of incompletely spliced HIV-1 RNAs that fail to become polyadenylated are likely retained in the nucleus and degraded.

Once the Rev/Crm-1/RanGTP complex assembles on the appropriate RNA, its journey from the site of synthesis to the cytoplasm begins. Most of the details of this process remain unclear but recent experiments have begun to identify host factors that play pivotal roles in the process. At least two members of the DEAD box RNA helicase family, DDX3 and DDX1, play essential roles in mediating Rev-dependent RNA export [113,114]. Depletion of either protein is associated with a marked reduction in Rev activity [113,114]. DDX1 also interacts with Rev via the N-terminal domain, suggesting a role in initial complex assembly [114]. For DDX3, its interaction with CRM-1 and localization to the outer nuclear membrane suggests that it might act to facilitate the translocation of the Rev-RNA complex through the nuclear pore [113]. Once on the cytoplasmic face of the nuclear membrane, another host factor, hRIP, appears to be required for release of the viral RNA into the cytoplasm [115,116] as depletion of the protein results in accumulation of viral RNA on the cytoplasmic face of the nucleus. A similar perinuclear accumulation of viral RNA is also observed upon overexpression of a C-terminal deletion mutant of Sam68, designated Sam68ΔC [83]. However, in this instance subsequent studies (Marsh and A.C., unpublished) indicate that this factor acts at a later step in the cytoplasmic metabolism of the viral RNA.

### CTE pathway

Although the study of the HIV-1/lentivirus systems clearly demonstrated a role for Rev-like proteins as adaptors to facilitate the export of viral RNAs to the cytoplasm, parallel work indicated that other viruses evolved alternative solutions to the export problem. An element at the 3' end of the MPMV designated the constitutive transport element (CTE) was shown to support Rev-independent HIV structural protein expression [117-119]. An element with similar activity was found in simian retrovirus type I [120]. The CTE is able to competitively inhibit cellular mRNA export (unlike Rev or the RRE) and interacts with the host export factor NXF1 [121-123]. Thus, in contrast to HIV-1 where Rev serves as an adaptor to access the export pathway, direct binding of NXF1 to the CTE bypasses the requirement for a virally-encoded protein.

### DR1/DR2 elements of avian retroviruses

Identification of the CTE export element in MPMV implied that similar export elements would be found in other simple retroviruses. One such element required for cytoplasmic accumulation of Pr-C RSV unspliced RNA was localized to the direct repeat region downstream of the *src *gene (DR2) [124]. A second repeat (DR1) located upstream of *src *shows similar activity, and at least one DR is required for RSV replication. The DR sequence is conserved between avian retroviruses and a similar activity was ascribed to the DR element in RAV-2 ALV [125]. Despite the clear export activity of the DR elements in reporter assays, unambiguous demonstration of an export role in RSV is complicated by the finding that DR deletions have effects apart from export, including destabilization of unspliced RNA and defective particle assembly [124,126,127]. While the DR elements harbor export activity, they are not absolutely required for export since the results from Simpson et al. [126] indicate that some unspliced RNA is exported and translated even in their absence.

As discussed above, HIV-1 Rev serves as an adaptor to target HIV RNA to the CRM1 export pathway, whereas the MPMV CTE directly binds NXF1 for export via the mRNA route. There is no obvious sequence similarity between the MPMV CTE and the avian DR elements, but it is perhaps reasonable to speculate that other simple retroviruses evolved to exploit the NXF1 pathway for export. Two studies took advantage of reagents to block the CRM1 pathway and found that, like the MPMV CTE, export of DR reporter RNAs was unaffected under conditions that blocked Rev export [125,128]. Thus, the CRM1 pathway is not required for ALV RNA export. Surprisingly, NXF1 binding to the ASV DR elements has yet to be demonstrated, implying that avian retroviral unspliced RNA export exploits yet a different pathway from HIV-1 and MPMV. However, the possibility that the avian DR elements bind a cellular adaptor molecule that functions similarly to NXF1, or that interacts with NXF1, has not been eliminated.

### The importance of the road traveled to the cytoplasm

It is well established that the nuclear history of an mRNA can influence its fate in the cytoplasm. This property can be attributed to the nature of the mRNP that assembles on the RNA. One well-studied example is the affect that mRNA splicing has on mRNP composition through the deposition of the exon junction complex (EJC) and its consequences to downstream events such as export, translation, and decay [129]. The possibility that cis elements that direct retroviral RNAs to one export pathway or another might influence downstream cytoplasmic events was realized with the observation that unspliced RSV RNAs that lack DRs produce readily detectable amounts of Gag protein but are defective in particle assembly [126,127]. These investigators hypothesized that either the RNA export defect rendered Gag synthesis below a threshold level required for assembly or more intriguingly, that the DR contains an element distinct from that responsible for CTE activity and directs RNA to a cytoplasmic location that is conducive to production of assembly-competent Gag protein.

Mammalian cells are nonpermissive for ALV infection due to defects in RNA processing, RNA export, Gag cleavage and particle assembly [124,126,130,131]. These observations are similar to those reported for ΔDR viruses in avian cells and suggest that the RNP exported in mammalian cells fails to deliver genome-length RNA to a cytoplasmic location where translated Gag can assemble particles. [124,126]. This idea is supported by work demonstrating that ALV particles can be formed in mammalian cells when the RRE is provided in cis and Rev in trans, i.e., when RNA is exported via the CRM1 pathway [106]. This result seems at odds with the lack of an effect of CRM1 inhibitors in avian cells, but it is possible that productive export occurs by more than one pathway, as is true for HIV-1 (see below).

A recent report by Swanson et al. demonstrated a similar link between HIV-1 unspliced RNA export and Gag assembly [132]. Gag protein can be produced in murine cells but is not processed or assembled into virions, which is one reason that murine cells are nonpermissive for HIV-1 replication. These investigators demonstrated that rerouting unspliced RNA export from the Rev/RRE pathway to the CTE pathway restored efficient virion production. This correlated with a redistribution of Gag from diffuse cytoplasmic localization when RNA export was Rev/RRE-dependent to plasma membrane association when the CTE route was used. Particle assembly occurs in human cells regardless of the export pathway used by the Gag RNA. Thus, as with ALV, productive HIV-1 RNA export may produce some type of RNP 'mark' that influences cytoplasmic RNA localization and the ability of the encoded protein to reach assembly sites. Part of this mark could lie in the association of hnRNP proteins on the unspliced RNAs at a particular step of the viral gene expression phase. Bériault et al. showed that disruption of hnRNP A2 binding to its cognate cis sequence (the hnRNP A2 response element or A2RE) also affected the cellular distribution of Gag and the auxiliary protein Vpr, but only at a late step that coincided with a block in unspliced HIV-1 RNA export from the nucleus [133]. It will be important to determine the composition of the RNPs that are and are not competent to direct productive Gag synthesis. This is clearly an area that deserves more research as it likely represents an interface between nuclear events and the formation/function of possible intracellular transport granules.

### Intracytoplasmic trafficking of retroviral RNA

Once delivered into the cytoplasm, two fates exist for the unspliced, genomic viral RNA: translation to produce the structural proteins and selection for encapsidation into the forming virions. The majority of unspliced retroviral RNA is not captured for encapsidation but serves other roles in generating viral structural proteins and enzymes or as a cofactor for assembly ([134-137] and reviewed in [138]). However, genomic viral RNA that is translated in the cytoplasm must transition in some fashion to sites of virus assembly to become encapsidated. The first evidence suggesting a specific location for genomic RNA selection for encapsidation has recently come to light in work from Andrew Lever's group. Using fluorescence resonance energy transfer (FRET), they were able to monitor the interaction of Gag with unspliced viral RNA. Unexpectedly, the unspliced HIV-1 RNA was found to be captured by Gag at a site at or adjacent to the centriole, near the nuclear membrane [139]. The signal was dependent upon psi-containing viral RNA. This was an infrequent event, consistent with earlier reports indicating that the vast majority of the unspliced viral RNA is not selected for encapsidation but translated or used as a cofactor for assembly [140,141]. The centriole region has also been identified as an assembly site for type D assembling retroviruses such as MPMV [142,143]. Image analysis reveals translating polyribosomes and co-assembly of capsids in this region but it remains unclear if assembly of retroviral type C HIV-1 capsids are also initiated in this region. This binding event could represent the first step in the formation of an RNP transport complex (see below) to sites of capsid formation. FRET has also been used successfully to identify cellular regions at which Gag-Gag homo-oligomerization in membranes occurs during viral assembly [144,145] and these types of techniques will help decipher some of the molecular interactions during viral replication.

Deciphering the relationship between the centriole, Gag capture and encapsidation into virions, and the process that directs viral genomic RNA (and possibly other viral mRNAs) to the sites of assembly remains a considerable challenge requiring targeting signals in the viral RNA, viral proteins, and/or a host cell targeting machinery [146]. While RNA can move intracellularly by a variety of mechanisms (Brownian motion, active transport) [147], clues about this process for retroviruses were provided by pioneering RNA trafficking studies in which vesicular trafficking pathways were shown to deliver viral components to assembly sites. Part of this newly described retroviral RNA trafficking pathway relies on the recruitment of genomic RNA from a cytoplasmic pool onto vesicles.

### Retroviral RNA trafficking on cellular vesicles in the cytoplasm

The movement of MLV RNA to sites of virion assembly was investigated by monitoring MLV genomic RNA movement in live cells using a bacteriophage MS2 tethering system. In this study [148], it was shown that genomic RNA traffics on recycling endosomal vesicles. Time-lapse fluorescence video microscopy showed a directed and linear trafficking pathway that was dependent upon the integrity of the microtubules [148]. Transport required the *psi *RNA packaging signal within the affected RNA and an intact NC domain in Gag, consistent with their demonstrated requirements for viral RNA packaging. The vesicles were comprised of both endosomal and lysosomal vesicles as evidenced by co-trafficking of the labeled RNA on transferrin- and lysotracker-positive vesicles in cells. Results of experiments in which monensin was used, a drug that prevents acidification of endosomal and organellar compartments, indicated that trafficking was achieved on vesicles that likely emanate from a steady-state endosomal compartment and not rapidly recycling vesicles that contain Rab11. Gag protein is recruited from late endosomal/lysosomal compartments to these endosomal membranes by the viral glycoprotein, Env, demonstrating important contributions of Env to this process. Not only could cellular RNAs replace viral RNA on vesicles in their system when psi was mutated, but some evidence suggests that the psi RNA sequence that is comprised of four stem loops in MLV, harbours an endosomal trafficking signal [149]. Other studies suggested a similar role for recycling membrane compartments and the expression of Env in the assembly of MPMV virus particles [143], although RNA trafficking was not examined. Despite the classical differences in the assembly pathways used by the two viruses (MLV is a type C virus for which capsid assembly and morphogenesis occur at the plasma membrane while MPMV capsids assemble intracellularly at the centriole [143]) the similarities in intracellular trafficking pathways that rely on recycling membrane compartments and the requirement for Env in the assembly of virus particles warrants further investigation [143]. Our own results add to this story with the demonstration that Env expression can dramatically alter the distribution of HIV-1 genomic RNA in HeLa cells (K. Lévesque, M. Halvorsen & A.J.M., unpublished). Basyuk and colleague's work supports earlier evidence that several retroviral Gags interact with kinesin motor proteins to enable trafficking along microtubules. The significance of these observations is yet to be deciphered but might put Gag as the key component of these trafficking complexes. Both Gag and RNA were visualized on the outside of translocating vesicles in the absence of MLV capsids, suggesting that part of this trafficking pathway is preceded by the formation of a cytosolic RNP complex [148]. While it in not known where the recruitment of MLV Gag and RNA occur, some insight was provided by the work of Poole's et al. in which HIV-1 RNA capture by Gag was shown to occur at the centriole [139]. MPMV RNA appears to be cotranslationally assembled in this cellular region, suggesting that this may be a point where the viral RNA transitions from a free RNP particle into a membrane-bound complex en route to the plasma membrane. It remains to be determined whether these relationships hold true for all retroviruses.

### The RNA trafficking granule takes shape

The directed movement of viral RNA within the cytoplasm relies on its interaction with multiple host proteins generating an RNA transport granule (RTG). The concept of an RTG derives from the studies in neuronal cells, which have both specialized functions and extended morphologies [150-152]. RTGs were shown to contain translational components such as transfer-RNA synthetases, EF1α, ribosomal RNAs, and molecular motor proteins such as dynein and kinesin [153] (Table 1). Although characterized in specialized neuronal cells, the RTG likely exists in some form in most other cell types, such as in fibroblasts, T cells and epithelial cells [154,155], but the morphologies of some cell types make it difficult to study RNA trafficking events (e.g., T cells). The RTG is indeed assembled in oligodendrocytes and each granule can contain multiple copies of HIV-1 mRNAs [151].

**Table 1:** Links between components of RNA trafficking granules and retroviral replication

RTG Component	Cellular Function	Link to Retroviral Replication Cycle	Virion-Incorporation	Reference*
Actin	Cytoskeletal component for cell structure; scaffold for intracellular trafficking	Acts with HIV-1 Rev to promote nucleo-cytoplasmic RNA Transport; Binds retroviral Gags	Yes	[178–181]
DDX1, 3, 5	RNA Helicases involved in RNA splicing, nuclear RNA export, RNA translation)	Promotes genomic RNA Nucleocyto-plasmic export	?	[113]
EF1α	Translation elongation factor involved in RNA anchoring via the actin cytoskeleton and in RNA translation	Binds HIV-1 Gag and may inhibit Gag RNA translation to favour encapsidation	Yes	[182]
eIF5A	Translation initiation factor involved in RNA translation initiation	A cofactor in Rev-mediated nucleocyto-plasmic RNA transport	?	[178, 183]
hnRNP A/B	Family of pre-mRNA splicing factors involved in RNA Splicing, RNA nucleocytoplasmic export, RNA trafficking	Influences retroviral RNA pre-mRNA splicing, RNA trafficking and gene expression	No	[34, 64, 133, 151]
Hsp70	Heat shock protein serving as a protein chaperone following heat or cellular stress	Promotes viral assembly, binds HIV-1 Vpr and promotes pre-integration complex nuclear nuclear import	Yes	[184–187]
Kinesin-1** (KIF-5, KHC)	Molecular motor protein involved in energy-dependent intracellular translocation	The kinesin, KIF-4, interacts with retro-viral Gag Proteins	?	[168, 169]
Nucleolin	RNA binding or chaperone protein with numerous nuclear functions: remodeling of chromatin structure, ribosomal DNA transcription, ribosomal RNA maturation, ribosome assembly and nucleocytoplasmic transport	Binds genomic RNA of MLV and HIV-1	Yes (MLV)	[188, 189]
PABP1	Poly(A) tail binding protein involved in RNA translation and RNA stability	Binds HIV-1 instability (INS) RNA sequences	?	[190]
PSF	Pre-mRNA splicing factor associated to polypyrimidine tract binding protein; Co-transcriptional RNA splicing	Interacts with HIV-1 cis-repressor (CRS) or INS RNA sequences	?	[191]
Pur1α	DNA- and RNA-binding protein involved in transcription and RNA transport	Binds TAR/Tat and can transactivate the HIV-1 LTR	?	[192–194]
RHA	RNA Helicase involved in RNA splicing and nuclear RNA export	Nucleocytoplasmic export and translation of genomic RNA	?	[156, 157] & K. Boris-Lawrie, personal communication
Staufen1***	Double-stranded RNA-binding protein involved in RNA trafficking & metabolism	Binds pr55^Gag ^and is in the genomic RNA ribonucleoprotein complex	Yes	[172, 175]
tRNA synthetases	Enzyme catalyzing the synthesis of tRNAs for RNA translation	Binds retroviral Gag and is virion incorporated	Yes	[195, 196]

* Proteins identified in the RTG reported in the following references: [160, 197]; ** related kinesin molecular motor protein; *** HIV-1 pr55^Gag ^should be considered part of a putative HIV-1 RTG since it is found in the Staufen1 complex ([172] and M. Milev & A.J.M., unpublished).

Published reports provide ample evidence that components of the RTG play roles in multiple steps of retroviral replication including transcription, RNA splicing, nucleocytoplasmic transport, translation as well as genomic RNA encapsidation (see Table 1). The effect of these factors on RNA transport within neuronal cells and the identified roles for many of these proteins in retrovirus replication highlight a potential functional relationship between the RTG machinery and retroviral replication. Retroviruses might co-opt components of this cellular machinery to ensure both the correct trafficking and localization of the retroviral RNA for presentation to the translation machinery and at sites of viral assembly for encapsidation into virions [146]. The proteins found in the RTG are presented in Table 1 and a subset are discussed below.

### RNA helicases: RHA, DDX1 & DDX3

As described above, nuclear export of retroviral RNA involves several cellular RNA helicases. Recent observations have identified roles for RNA helicase A (RHA) and two DEAD-box proteins, DDX1 and DDX3 in the nuclear export of retroviral RNAs [113,156-158]. The functions of the latter proteins have been reviewed earlier [159] and are described in a previous section of this review. These RNA helicase proteins appear to act at different stages of retroviral gene expression. RHA depletion by siRNA decreases translation of HIV-1 *gag-pol *mRNA, perhaps by disrupting the remodeling of RNA-RNA and RNA-protein interactions that are required for usage of unspliced transcripts by the translation apparatus (K. Boris-Lawrie, unpublished results). The recent identification of these proteins in RTGs in the cytoplasm [160], albeit in specialized neuronal cells, leads to the idea that they may represent part of the "protein mark" that initially tags retroviral RNAs in the nucleus and remains associated during nuclear export and subsequent RTG assembly in the cytoplasm. These findings suggest a possible mechanism by which the nuclear history of the retroviral RNA and the associated proteins might dictate gene expression patterns in the cytoplasm analogous to the role of the exon junction complex [161,162].

### hnRNP A2

hnRNPs in general are believed to function at many post-transcriptional levels (reviewed in [163]). Particular attention has been placed on their role in the retroviral RNA processing and nuclear export [39,64,164,165]. Some of the evidence that particular hnRNPs could play other -yet related- roles in retroviral replication such as RNA trafficking are outlined here. Two 21 nucleotide sequences in HIV-1 RNA were shown to bear striking homology to the myelin basic protein (MBP) A2RE located in the 3' untranslated region of the corresponding mRNA. The MBP A2RE is required for RNA trafficking to the extremity of dendrites of murine oligodendrocytes [150,166]. Injected HIV-1 and HIV-2 RNAs are also efficiently trafficked in these cells with requirements identical to those described for MBP mRNA: an intact A2RE, hnRNP A2 expression, microtubules and kinesin ([151] & A.J.M, E. Barbarese, É.A. Cohen, J. Carson, unpublished results). By sequence comparison, similar cis-acting elements can also be found in HTLV-1 and HIV-2 RNAs but only the element in HIV-2 *vpr *RNA has been shown to be active in RNA trafficking [151]. Subsequent studies using proviral HIV-1 constructs confirmed these results and demonstrated that hnRNP A2 association with A2RE is critical for late viral gene expression and genomic RNA encapsidation [133]. Kinesin motor proteins are critical for hnRNP A2-mediated RNA trafficking in oligodendrocytes and hnRNP A2 was recently shown to interact with microtubule adaptor proteins [167] and is present in RNA transport granules [160]. This observation provides a physical link to the cytoskeleton on which organelles, vesicles, and RTGs are transported. The interactions between retroviral Gag proteins and the kinesin motor protein, KIF-4, also suggest that large multi-protein complexes are translocated on the microtubule-based cytoskeleton in virus-expressing cells [168,169]. These examples provide evidence that viral RNP translocation is dictated by the activities of a variety of viral and cellular proteins.

### Staufen1

While many of the proteins within the RTG have functions in several cellular compartments, Staufen1 works mainly in the cytoplasm. Staufen1 represents a *bona fide *RNA trafficking protein whose function is conserved from lower to higher eukaryotes. It plays a critical role in the localization of several mRNAs in *Drosophila*, likely via its direct interaction with target RNAs or in the context of RNP complexes [170,171]. Several lines of evidence implicate Staufen1 in regulating retroviral genomic RNA encapsidation. First, Staufen1 associates with precursor Gag protein and not the mature Gag proteins, and preferentially co-precipitates with the genomic viral RNA but not spliced forms [172]. Second, Staufen1 is encapsidated in virions in stoichiometry to the number of genomic RNA molecules present. Its identification in other retrovirus particles suggests that Staufen1 may have a more general role in the selection of genomic RNA for retroviral encapsidation. Third, *Drosophia Staufen* preferentially binds RNA homodimers [173], the form viral genomic RNA takes during virion morphogenesis and within retrovirus particles [174]. Fourth, Staufen1 knockdown by siRNA results in a dramatic decrease in infectivity and virus production [172]. Fifth and more importantly, modulation of intracellular levels of Staufen1 directly impacts on genomic RNA encapsidation [172,175]; L. Abrahamyan, J.-F. Clément & A.J.M., unpublished results). These findings suggest that Staufen1 may tag the genomic RNA for encapsidation in the cytoplasm and be concomitantly recruited into virus particles. Its function in this process needs to be unequivocally proven, however published data suggest that a molecular switch could be at play that promotes the selection of two copies of genomic RNA per virion [172]. Likely, the activity of Staufen1 is but one of several factors needed for the "genomic RNA selection" process. It will be important to evaluate how the functions of this and other RNA trafficking proteins are interrelated along the retroviral RNA targeting pathway from the nucleus to progeny virions.

## Conclusion

Several functional and physical links between the transcription, RNA processing, nucleocytoplasmic and cytoplasmic transport machineries as well as the metabolic machineries for RNA in the cytoplasm are made by RNA-binding proteins, some of which mark the RNA before their exit from the nucleus and modulate RNA fate in the cytoplasm. The journey of retroviral RNA from the nucleus to its ultimate destination in the capsid is likely to be characterized by similar phenomena, yet up until now the details of these events have been scant. Many details of how the RNA processing machinery is manipulated to produce the appropriate spectrum of mRNAs and to allow genome-length RNA to escape splicing have been revealed in the last few years and illuminated potential targets for arresting virus replication. Recent work has already exploited our understanding of retroviral RNA processing to develop small molecule inhibitors of HIV-1 RNA splicing [176]. Another critical step in replication is the nuclear export of genome-length RNA and efforts are being made towards gaining a better understanding of the processes that remodel nuclear RNPs and determine the fates of viral RNA within the cytoplasm. A number of factors with roles in cytoplasmic RNA transport have been found associated with retroviral mRNA. A more detailed understanding of the formation and function of retroviral RNA trafficking granules and their components may provide new insights into targets for rationale drug design, an idea that recently has met with some success [177].

## Competing interests

The author(s) declare that they have no competing interests.

## Authors' contributions

All authors contributed equally to the inception and writing of the manuscript.
